# Heterotrimeric G-Protein Signaling Is Required for Cellulose Degradation in Neurospora crassa

**DOI:** 10.1128/mBio.02419-20

**Published:** 2020-11-24

**Authors:** Logan A. Collier, Arit Ghosh, Katherine A. Borkovich

**Affiliations:** a Department of Microbiology and Plant Pathology, University of California, Riverside, Riverside, California, USA; b Graduate Program in Biochemistry and Molecular Biology, University of California, Riverside, Riverside, California, USA; Duke University

**Keywords:** G proteins, cell signaling, cellulolytic enzymes, cyclic AMP, filamentous fungi, lignocellulose, molecular genetics

## Abstract

The filamentous fungus Neurospora crassa decomposes lignocellulosic biomass to generate soluble sugars as carbon sources. In this study, we investigated a role for heterotrimeric G-protein signaling in cellulose degradation. Loss of the Gα subunit genes *gna-1* and *gna-3*, the Gβ subunit genes *gnb-1* and *cpc-2*, the Gγ gene *gng-1*, or the gene for downstream effector adenylyl cyclase (*cr-1*) resulted in loss of detectable cellulase activity. This defect was also observed in strains expressing a constitutively active version of *gna*-*3* (*gna-3^Q208L^*). We found that GNA-1 levels are greatly reduced in Δ*gna*-*3*, Δ*gnb-1*, and Δ*gng-1* strains, likely contributing to cellulase defects in these genetic backgrounds. The observation that *gna-3^Q208L^* Δ*gnb-1* strains exhibit cellulase activity, despite greatly reduced levels of GNA-1 protein, is consistent with positive control of cellulase production by GNA-3 that is manifested in the absence of *gnb-1*. Expression patterns for five cellulase genes showed that Δ*gna-1*, Δ*gnb-1*, and Δ*gna-3* mutants produce less cellulase mRNA than the wild type, consistent with transcriptional regulation. Δ*cpc-2* mutants had wild-type levels of cellulase transcripts, suggesting posttranscriptional control. In contrast, results for Δ*cr-1* mutants support both transcriptional and posttranscriptional control of cellulase activity by cAMP signaling. Cellulase activity defects in Δ*gna-3* mutants were fully remediated by cAMP supplementation, consistent with GNA-3 operating upstream of cAMP signaling. In contrast, cAMP addition only partially corrected cellulase activity defects in Δ*gna-1* and Δ*gnb-1* mutants, suggesting participation of GNA-1 and GNB-1 in additional cAMP-independent pathways that control cellulase activity.

## Abstract (precis)

Filamentous fungi are critical for the recycling of plant litter in the biosphere by degrading lignocellulosic biomass into simpler compounds for metabolism. Both saprophytic and pathogenic fungi utilize plant cell wall-degrading enzymes to liberate carbon for metabolism. Several studies have demonstrated a role for cellulase enzymes during infection of economically relevant crops by fungal pathogens. Especially in developing countries, severe plant disease means loss of entire crops, sometimes leading to starvation. In this study, we demonstrate that G-protein signaling is a key component of cellulase production. Therefore, understanding the role of G-protein signaling in the regulation of the unique metabolism of cellulose by these organisms can inform innovations in strain engineering of industrially relevant species for biofuel production and in combatting food shortages caused by plant pathogens.

## INTRODUCTION

The cell walls of all plants are primarily composed of lignocellulosic biomass, making it the most abundant biological composite produced on earth (1). In agricultural processes, this material is considered a waste by-product, but it is commonly used as feedstock for the production of alternative fuel sources (2, 3). Secreted enzymes from filamentous fungi are used to degrade lignocellulose polymers. Optimizing fungal platforms for greater enzyme production is a major bottleneck to cost-efficient biofuels (4). Additionally, numerous fungal species are plant pathogens that decimate crops (5), and several studies have demonstrated a role for cellulase enzymes during infection (reviewed in reference 6). Understanding the pathways of carbon sensing in these organisms will have significant implications in the optimization of agricultural methods and fuel production.

Fungi differentially regulate enzymes for the catabolism of specific carbon polymers. For example, in Neurospora crassa and Aspergillus nidulans, cellulose-specific regulons have been well characterized (7). Studies in Trichoderma reesei, several *Aspergillus* species, and N. crassa indicate that cellobiose, a breakdown product of cellulose, is the inducer of cellulolytic genes (8), demonstrating that some cellulose deconstruction is required prior to full cellulase induction. In many fungal species, glucose sensing activates the transcription factor CRE-1, preventing the induction of cellulolytic genes in a process called carbon catabolite repression (CCR) (2, 7, 9). Derepression of CRE-1 allows expression of the transcription factor CLR-1, leading to transcription of CLR-2, which activates transcription of most cellulolytic genes (7, 10, 11).

Genes that are induced via the disruption of the CCR response and the presence of cellobiose are part of the plant cell wall degradation network (12). In this network, carbohydrate-active enzymes (CAZymes) that degrade cellulose are termed cellulases. These can be categorized into several classes, including cellobiohydrolases, endoglucanases, β-glucosidases, polysaccharide monooxygenases, and cellobiose dehydrogenases, each of which play a specific role in cleaving β-1,4-linkages present in cellulose (reviewed in reference 1).

Filamentous fungi use heterotrimeric G proteins and membrane-associated G-protein-coupled receptors (GPCRs) to sense and respond to their environment (13, 14). G proteins consist of α, β, and γ subunits, with the βγ subunits tightly associated (15–17). G-protein signaling begins with GPCR activation by a ligand, causing the GPCR to facilitate exchange of GDP for GTP on the Gα subunit. This causes dissociation of the Gα from the βγ dimer, allowing both to signal downstream effectors. N. crassa uses G proteins to regulate basal hyphal growth, sexual development, asexual sporulation, and stress responses (13, 18–23). N. crassa possesses three Gα proteins (GNA-1, GNA-2, and GNA-3) (24), two Gβ proteins (GNB-1 and CPC-2) (25), and a Gγ that associates with GNB-1 (GNG-1) (26). N. crassa also has two protein kinase A (PKA) catalytic subunits (27, 28) that are predicted downstream targets of cAMP produced by the adenylyl cyclase CR-1 (29). Previous studies have shown that CR-1 is an effector of G-protein signaling in N. crassa, with GNA-1 regulating GTP-stimulated CR-1 enzyme activity (30) and GNA-3 controlling CR-1 protein levels (21).

The three N. crassa Gα subunits are required for carbon sensing (22, 31) but have not been implicated in cellulose degradation. In *T. reesei*, constitutive activation of the *gna-3* homolog results in cellulase expression in constant light (32), while deletion of the homologous *gna-1* gene leads to increased cellulase expression in darkness (33). In A. nidulans, deletion of *pkaA* results in an inability to localize CreA to the nucleus, causing misregulation of CCR and induction of hydrolytic enzymes on glucose medium (11). Recent work in N. crassa and Myceliophthora thermophila showed that the CLR-4 transcription factor binds to the promoter region of *cr-1* in both fungi. Loss of *clr-4* resulted in downregulation of *cr-1* and cellulase genes, and strains overexpressing the *cr-1* open reading frame exhibited a 3- to 20-fold increase in cellulase mRNA levels (34), implicating cAMP signaling in the regulation of cellulase gene expression.

In a recent study, we analyzed phenotypes for mutants lacking 21 of the known Pth11-like GPCRs in N. crassa. Three mutants exhibited growth phenotypes when cultured on medium containing Avicel (crystalline cellulose) (35). Pth11 is required for pathogenicity on rice (Oryza sativa) by Magnaporthe oryzae (36). This GPCR physically interacts with MagA (a Gα homologous to GNA-3) and adenylyl cyclase during early stages of pathogenesis (37). A recent investigation showed that two Fusarium graminearum Pth11-like proteins required for virulence against wheat interact with Gα proteins when expressed in yeast (38). These findings support an interaction between Pth11-like GPCRs and heterotrimeric G proteins in filamentous fungi.

Our previous work showing Pth11-like GPCRs influence growth of N. crassa on Avicel prompted investigation into the role that G proteins play in cellulose sensing and degradation in N. crassa. In this study, we embarked on a systematic analysis of cellulose phenotypes for all known G protein subunits and adenylyl cyclase. For this work, we utilized deletion mutants for all six G protein subunits (24, 25, 39) and strains expressing constitutively activated GTPase-deficient versions of each Gα subunit gene (40) (*gna-1^Q204L^*, *gna-2^Q205L^*, and *gna-3^Q208L^*) (see Table S1 in the supplemental material). Our results provide evidence for transcriptional, posttranscriptional, and cAMP-dependent and -independent control of cellulase enzymes via G-protein signaling in N. crassa.

10.1128/mBio.02419-20.1 TABLE S1 Strains used in this study. FGSC, Fungal Genetics Stock Center. Download Table S1, DOCX file, 0.03 MB. Copyright © 2020 Collier et al. 2020 Collier et al. This content is distributed under the terms of the Creative Commons Attribution 4.0 International license.

## RESULTS

### Alteration of growth, cellulase activity, and supernatant protein concentration in mutants cultured on Avicel.

We began our analysis of roles for G proteins in cellulose degradation by growing strains in shaken liquid cultures containing Avicel using a previously described approach (41). This method relies on the observation that intact Avicel is insoluble and will sediment at the bottom of cultures after centrifugation. After 3 days, wild-type, Δ*gna-1*, and Δ*gna-2* strains appeared to have degraded the Avicel (see Fig. S1 in the supplemental material). In contrast, cultures of Δ*gna-3*, Δ*gnb-1*, Δ*gng-1*, and Δ*cpc-2* mutants contained residual Avicel, suggesting that these G protein subunits are required to degrade cellulose into soluble cellodextrins in N. crassa.

10.1128/mBio.02419-20.4 FIG S1 Growth of strains after direct inoculation into Avicel medium. N. crassa strains were grown at 25°C in constant light for 4 days in 25 ml of VM containing 2% Avicel as the carbon source (see Text S1). Strains that did not completely degrade Avicel into soluble glucose or glucose oligomers still have Avicel (white powder) remaining in the bottom of the tube. The black line indicates the top of the Avicel pellet. There was no remaining Avicel pellet in cultures from the wild type or Δ*gna-1* or Δ*gna-2* mutants. Download FIG S1, PDF file, 0.07 MB. Copyright © 2020 Collier et al. 2020 Collier et al. This content is distributed under the terms of the Creative Commons Attribution 4.0 International license.

10.1128/mBio.02419-20.3 TEXT S1 Supplemental methods. Download Text S1, DOCX file, 0.04 MB. Copyright © 2020 Collier et al. 2020 Collier et al. This content is distributed under the terms of the Creative Commons Attribution 4.0 International license.

Mutants for both of the known PKA catalytic subunits in N. crassa were tested in preliminary experiments, with Δ*pkac-1* mutants containing residual Avicel during growth and Δ*pkac-2* mutants resembling the wild type. These observations were supported by preliminary measurements of cellulase activity in cell-free supernatants from these two mutants. These results suggested that while PKAC-1 may be more important with regard to cellulase activity, there is some redundancy between the two catalytic subunits. We attempted to create a double mutant lacking both PKA catalytic subunit genes (28) but were unsuccessful. Therefore, we utilized the Δ*cr-1* mutant, which lacks intracellular cAMP (29, 41), to approximate the loss of cAMP signaling in our study. The results showed that Δ*cr-1* mutant cultures contained residual Avicel, implicating adenylyl cyclase and cAMP signaling in the degradation of cellulose.

The most likely explanation for the reduced tissue formation observed in most of the mutants is a reduced accumulation of cellulase enzymes. To address this possibility, we measured cellulase activity and protein concentration in cell-free supernatants from the strains. To help mitigate the mass accumulation issues observed after direct inoculation into Vogel’s minimal medium (VM)-Avicel described above, we pregrew cultures in VM-glucose for 16 h and then transferred them to VM-Avicel or to VM-glucose for 3 days. We then collected cell-free supernatants for the assays and extracted and quantified the cell biomass protein in the cell pads. The cellulase activity and protein concentration in the supernatant samples were normalized to cell biomass protein, to account for possible growth defects due to the gene deletion.

**FIG 1:**
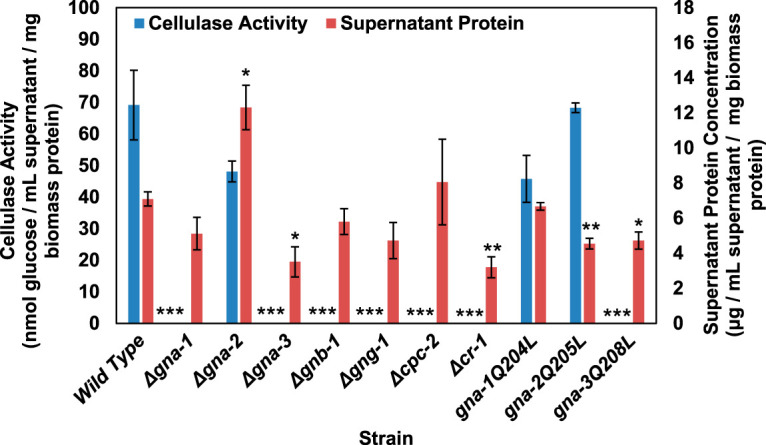
Cellulase activity and protein concentrations in culture supernatants after growth in VM-Avicel. Cultures were grown overnight (16 h) on VM-glucose and then washed twice in VM without a carbon source. The cultures were transferred to VM-Avicel or to fresh VM-glucose and grown for 3 days or overnight, respectively. A sample of the culture supernatant was withdrawn and passed through a 0.45-μm filter. Total protein was extracted from the cell pads of each culture as described in Materials and Methods. All protein concentrations were determined using the BCA protein assay. Cellulase activity was assayed as described in reference 44. A minimum of three replicates were used, and errors are expressed as the standard error. Statistical significance relative to wild-type *mat***a** was determined using a two-tailed Student’s *t* test, and strains with protein levels or cellulase activity significantly different from that of the wild type are indicated as follows: *, *P* < 0.05; **, *P* < 0.01; ***, *P* < 0.001.

We assessed cellulase activity in the cell-free supernatant samples using an *in vitro* coupled enzyme assay that detects glucose levels after incubation of samples with Avicel (41). Δ*gna-1*, Δ*gna-3*, Δ*gnb-1*, Δ*gng-1*, Δ*cpc-2*, Δ*cr-1*, and *gna-3^Q208L^* strains had no detectable cellulase activity (Fig. 1). The lack of cellulase activity in Δ*gna-1* culture supernatants contrasts with the ability of the mutant to degrade Avicel (Fig. S1). This may result from degradation of Avicel into smaller cellodextrins but not completely to glucose. This result shows that loss of five of the six G protein subunits or adenylyl cyclase has a profound effect on cellulase activity. The observation that loss and activation of *gna-3* result in similar phenotypes is reminiscent of the relationship between the Gpa1p Gα and the Ste4p Gβ in Saccharomyces cerevisiae (42–44). By analogy, this suggests a regulatory circuit involving tethering between GNA-3 and the Gβγ dimer in N. crassa.

Protein measurements demonstrated that only three of the strains with undetectable cellulase activity had reduced extracellular protein relative to that of the wild type after growth on Avicel: Δ*gna-3*, Δ*cr-1*, and *gna-3^Q208L^* (Fig. 1). Furthermore, the reduction in extracellular protein for these three strains (maximum ∼55%) does not explain the loss of detectable cellulase activity. In contrast to the strains with severe cellulase defects, we observed that Δ*gna-2* and *gna-2^Q205L^* strains had higher and lower levels, respectively, of extracellular protein on Avicel (Fig. 1). The opposing results obtained for loss versus activation of *gna-2* implicate GNA-2 as a negative regulator of supernatant protein accumulation on VM-Avicel.

To determine whether the protein accumulation defects observed in some strains on Avicel extended to growth on glucose, we compared the results for these two carbon sources (see Fig. S2). No strain had lower protein accumulation on glucose than the wild type, and levels were actually elevated in Δ*cpc-2* mutants. Δ*gna-3*, Δ*gnb-1*, Δ*gng-1*, Δ*cpc-2*, Δ*cr-1*, and *gna-3^Q208L^* strains accumulated less protein on VM-Avicel than on VM-Glucose. *gna-1^Q204L^* strains had a slight increase in protein accumulation on Avicel relative to that on glucose.

10.1128/mBio.02419-20.5 FIG S2 Protein concentration in culture supernatants after growth in VM-Avicel or VM-glucose. Cultures were grown overnight (16 h) on VM-glucose and then were washed twice in VM without a carbon source. The cultures were transferred to VM-Avicel or to fresh VM-glucose and grown for three days or overnight, respectively. A sample of the culture supernatant was withdrawn and filtered through a 0.45-μm filter to remove cells. Total protein was extracted from the cell pads of each culture (biomass protein) as described in Materials and Methods. The protein concentration in the filtered culture supernatants and the cell pad extracts was determined using the BCA assay. The culture supernatant values were then normalized by dividing by the biomass protein. A minimum of three replicates were used, and errors are expressed as the standard errors. Differences relative to the wild type on the same carbon source were determined using a two-tailed Student’s *t* test, and strains with protein levels significantly different than the wild type are indicated as follows: *, *P* < 0.05; **, *P* < 0.01. Strains with protein levels that differ significantly on the two carbon sources are indicated with an asterisk above the bracket spanning the VM-glucose and VM-Avicel values. Download FIG S2, PDF file, 0.06 MB. Copyright © 2020 Collier et al. 2020 Collier et al. This content is distributed under the terms of the Creative Commons Attribution 4.0 International license.

To address the possibility that impaired glucose transport contributes to the mass accumulation defects of some mutants on Avicel, we performed the glucose detection assay with culture supernatants from glucose-grown cultures. None of the strains in this study had detectable glucose remaining in their supernatants after 3 days of growth on VM-glucose, suggesting that either they have normal glucose transport or that 3 days is adequate for uptake of all glucose in the medium.

Previous work has demonstrated different protein banding patterns after subjecting cell-free supernatants of mutants impaired in cellulase production to SDS-PAGE (41, 45). To explore this possibility with our set of strains, we subjected equal volumes of the concentrated VM-Avicel supernatant samples to SDS-PAGE analysis (see Fig. S3A). Several prominent bands were observed in wild-type samples, with the most intense appearing at 70 kDa, the migration position of several cellulase enzymes (41). This band was excised from gels containing wild-type samples and subjected to liquid chromatography-mass spectrometry (LC-MS) (45). Among the identified proteins, cellobiohydrolase CBH-1 (NCU07340) was present in the largest amount (41% of the total) (Fig. S3B), followed by the β-glucosidase GH3-4 (NCU04952; 13%) and the non-cell wall-anchored protein NCW-1 (NCU05137; 12%).

10.1128/mBio.02419-20.6 FIG S3 Proteins in the 70-kDa region after SDS-PAGE of culture supernatants. (A) SDS-PAGE analysis of cell-free supernatants from VM-Avicel cultures. One milliliter of cell-free culture supernatant from each of the indicated strains was concentrated as described in Materials and Methods. A volume containing 20 μl was subjected to SDS-PAGE using a 10% resolving gel. The positions of molecular weight markers are shown on the left. The prominent band at 70 kDa is indicated by the red rectangle. Volumes of culture supernatant have not been adjusted for cell pad biomass. (B) Discovery proteomics of the protein band at 70 kDa. Aliquots containing 20 μg of protein from cell-free supernatants obtained from wild-type *mat***a** were electrophoresed on a 10% SDS-PAGE gel. Slices corresponding to the 70-kDa region were excised and analyzed using LC-MS as described in the Text S1. Proteins displayed in the table had a minimum of 10 peptide spectral matches (PSMs) in all three biological replicates. The number of PSMs for each protein was divided by the total detected in each sample to give the percent total PSMs. Errors are expressed as the standard errors. Download FIG S3, PDF file, 0.09 MB. Copyright © 2020 Collier et al. 2020 Collier et al. This content is distributed under the terms of the Creative Commons Attribution 4.0 International license.

Inspection of the stained SDS-PAGE gels showed that levels of the 70-kDa band were lowest in Δ*gna-1*, Δ*gna-3*, Δ*gnb-1*, and *gna-3*^Q208L^ strains (Fig. S3A), consistent with loss of cellulase activity (Fig. 1). Although Δ*gng-1*, Δ*cpc-2*, and Δ*cr-1* strains still produce a prominent band at 70 kDa (Fig. S3A), these strains lack detectable cellulase activity, suggesting that the cellulases are nonfunctional.

Overall, our results demonstrate that of the three Gα subunits, GNA-2 does not greatly influence cellulase activity. GNA-1 is required for activity, but constitutive activation does not lead to higher levels. Loss or activation of *gna-3* leads to reduced cellulase activity, suggesting a mechanism involving a tethering relationship between GNA-3 and the Gβγ dimer.

### Levels of the Gα GNA-1 are reduced in Δ*gna-3*, Δ*gnb-1*, and Δ*gng-1* mutants.

The above-described results show that most of the G protein subunits are required for N. crassa to efficiently degrade cellulose into glucose. We have shown previously that loss of the Gβγ dimer affects levels of Gα proteins and that mutation of *gna-1* or *gng-1* results in lower GNB-1 levels during growth on VM-sucrose (24, 26). To investigate whether this is also the case for VM-Avicel, we isolated membrane fractions from VM-Avicel-grown cell pads and used Western blotting to determine levels of the different G protein subunits. Since CPC-2 is cytoplasmic in N. crassa (25), whole-cell extracts were used to determine protein levels in the various strains.

**FIG 2:**
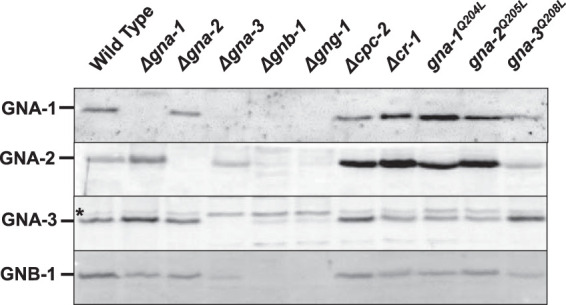
Levels of G protein subunits in strains cultured on Avicel medium. Whole-cell extracts were prepared from strains cultured on Avicel as described in Materials and Methods. Differential centrifugation was used to isolate the particulate fraction, which was then utilized to check amounts of the membrane-associated Gα proteins and GNB-1. Since CPC-2 is cytoplasmic (25), whole-cell extracts were used for determining CPC-2 levels. Samples containing equal amounts of protein were subjected to SDS-PAGE, and gels were blotted to nitrocellulose. Blots were reacted with antiserum for GNA-1, GNA-2, GNA-3, GNB-1, or CPC-2 (see Materials and Methods). The results shown are representative of three biological replicates. The migration position of each protein is indicated along the left. An asterisk indicates a background band that cross-reacts with GNA-3 antiserum.

The results from Western blot analysis demonstrate that as observed for sucrose, deletion of *gnb-1* or *gng-1* results in greatly reduced amounts of GNA-1 and GNA-2 and a slight decrease in GNA-3 levels during growth on Avicel (Fig. 2). Additionally, levels of GNA-1 were significantly reduced in Δ*gna-3* mutants. GNB-1 was not detectable in Δ*gng-1* mutants and was diminished in Δ*gna-3* strains. CPC-2 does not appear to be affected by the other G proteins, since levels were similar in all strains. Since GNA-1 and GNA-3 appear to positively regulate cellulase activity in N. crassa, reduced amounts of these Gα proteins may explain loss of cellulase activity in many strains (Fig. 1). Taken together, the results show that *gna-3*, *gnb-1*, and *gng-1* are most important for maintaining protein levels of the other subunits during Avicel growth.

### Constitutive activation of GNA-1 or GNA-3 completely or partially overrides the effects of the Δ*gnb-1* mutation.

**FIG 3:**
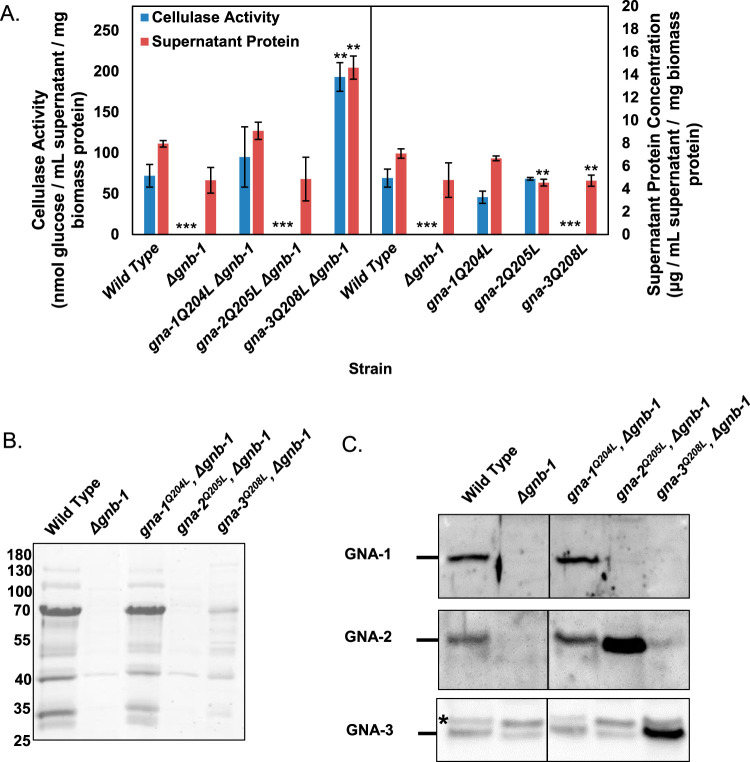
Analysis of strains containing activating Gα mutations in a Δ*gnb-1* background. Cultures were grown as described in the legend to Fig. 1. Both cell-free culture supernatants and cell pads were retained. (A) Cellulase activity and supernatant protein concentrations. Assays were performed as described in the legend to Fig. 1. Errors are expressed as the standard errors. Statistical significance relative to wild-type *mat***a** was determined using a two-tailed Student’s *t* test, and strains with cellulase activity or supernatant protein concentrations significantly different from that of the wild type are indicated as follows: *, *P* < 0.05; **, *P* < 0.01; ***, *P* < 0.001. Values from *gna-1^Q204L^*, *gna-2^Q205L^*, and *gna-3^Q208L^* were replotted from Fig. 1 for comparison. The vertical line on the graph indicates that the two groups of strains were analyzed separately. (B) SDS-PAGE analysis of supernatants. SDS-PAGE of supernatant protein from the indicated strains was performed as described in the legend to Fig. S3 in the supplemental material. (C) Levels of Gα subunits in strains containing constitutively active Gα mutations in a Δ*gnb-1* background. Differential centrifugation was used to isolate the particulate fraction from whole-cell protein extracts. Samples were subjected to SDS-PAGE and gels were blotted to nitrocellulose. Blots were reacted with antisera for GNA-1, GNA-2, and GNA-3. The results shown are representative of three biological replicates. The migration position of each protein is shown along the left.

The observation that Δ*gnb-1* mutants lack detectable cellulase activity is consistent with *gnb-1* as a positive regulator. However, the negative influence of the Δ*gnb-1* mutation on Gα protein levels (Fig. 2) suggested a more complex interaction. Furthermore, the absence of cellulase activity in the Δ*gna-3* and *gna-3^Q208L^* strains supports negative regulation by *gnb-1*. We therefore investigated whether any of the three constitutively active Gα alleles could override the defects of the Δ*gnb-1* mutation. We examined cellulase activity and extracellular protein levels in the strains as well as the banding pattern of supernatant proteins during SDS-PAGE analysis (Fig. 3A and B).

The Δ*gnb*-*1 gna*-*1^Q204L^* strain resembled the *gna*-*1^Q204L^* and wild-type strains with regard to cellulase activity (Fig. 3A). Additionally, the concentration and SDS-PAGE banding pattern of the extracellular proteins were similar to those of the wild type. These results are consistent with a scenario in which *gna-1* is epistatic to *gnb-1* (Fig. 3A and B).

Cellulase activity was not detected in the Δ*gnb*-*1* or Δ*gnb*-*1 gna*-*2^Q205L^* strains, while the *gna*-*2^Q205L^* strain was normal (Fig. 3A). Although levels of extracellular protein were normal in the Δ*gnb*-*1 gna*-*2^Q205L^* strain (Fig. 3A), the SDS-PAGE protein banding pattern resembled that of the Δ*gnb*-*1* strain (Fig. 3B). The inability of the *gna*-*2^Q205L^* allele to increase cellulase activity in the Δ*gnb*-*1* background supports GNB-1 operating downstream of GNA-2 (Fig. 3A and B).

In contrast to the nondetectable levels in the Δ*gnb*-*1* and *gna*-*3^Q208L^* strains, cellulase activity in the Δ*gnb*-*1 gna*-*3^Q208L^* strain was significantly greater than in the wild type (Fig. 3A). Likewise, extracellular protein levels were higher than in the wild type (Fig. 3A). The SDS-PAGE protein banding pattern resembled that of the wild type but was less intense (Fig. 3B). These results suggest a positive role for GNA-3 in regulating cellulase activity that is normally masked by a negative regulatory activity for GNB-1.

To investigate the epistatic relationships further, we performed Western blot analysis for GNA-1, GNA-2, and GNA-3 in this group of strains (Fig. 3C). We found that the only strain with detectable levels of GNA-1 was Δ*gnb*-*1 gna*-*1^Q204L^* (Fig. 3C). The low levels of GNA-1 in the Δ*gnb*-*1 gna*-*2^Q205L^* strain correlate with the lack of activity in this background. GNA-3 appears to be the only Gα present in the Δ*gnb*-*1 gna*-*3^Q208L^* strain (Fig. 3C). This last observation, coupled with the high cellulase activity in the Δ*gnb*-*1 gna*-*3^Q208L^* strain, further supports a model in which the positive action of GNA-3 on cellulase activity is impaired when GNB-1 is present.

### Cellulase mRNA levels are reduced in Δ*gna-1*, Δ*gna-3*, and Δ*gnb-1* mutants.

We next asked whether the defects observed in the single-gene deletion mutants resulted from reduced cellulase gene expression. We assessed mRNA levels at 4 h after transfer to Avicel, to reflect early fluctuations in CCR and cellulase mRNA induction. We selected five well-characterized cellulase genes that are among the most highly expressed in wild-type N. crassa (8) and encode the most abundant proteins in the cellulose secretome (45) for quantitative reverse transcriptase PCR (qRT-PCR). Three of the five genes also encode proteins that were constituents of the prominent 70-kDa band in wild-type protein samples after SDS-PAGE (Fig. S3). *cbh-1* (NCU07340) and *gh6-2* (NCU09680) encode exo-β-1,4-glucanases, while *gh5-1* (NCU00762) corresponds to an endo-β-1,4-glucanase (12). *gh3-4* (NCU04952) encodes the only β-glucosidase in the N. crassa genome with a predicted secretion signal (8). *cdh-1* (NCU00206) is the most highly expressed cellobiose dehydrogenase in N. crassa, and its deletion results in a 37% to 49% reduction in cellulase activity (46).

**FIG 4:**
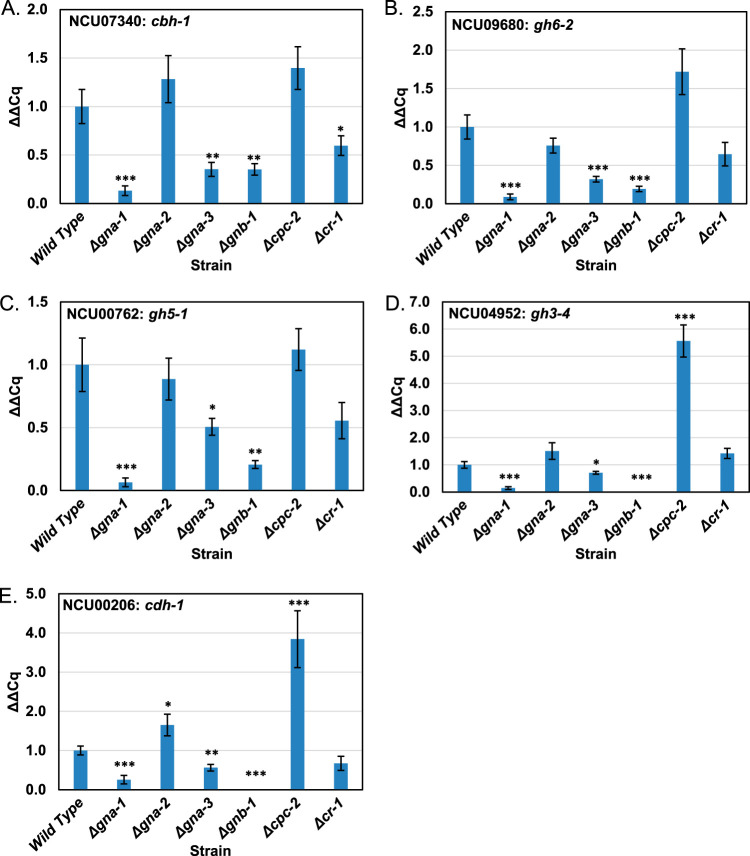
qRT-PCR to determine mRNA levels for major cellulases. Cultures were grown on VM-glucose for 12 h and then transferred to VM-Avicel for 4 h. Total RNA isolated from the indicated strains was then converted to cDNA using reverse transcriptase. The cDNA was then used as a template in qPCRs for *cbh-1*/NCU07340, *gh6-2*/NCU09680, *gh5-1*/NCU00762, *gh3-4*/NCU04952, *cdh-1*/NCU00206, and actin/NCU04173 (control). In all, three biological replicates were used, and three technical replicates were tested for each biological replicate. Values were normalized to actin and then to the wild type. Errors are expressed as the standard errors. *, *P* < 0.05; **, *P* < 0.01; ***, *P* < 0.001 versus wild type.

The results from qRT-PCR analysis demonstrated that relative to that in the wild type, Δ*gna-1*, Δ*gna-3*, and Δ*gnb-1* mutants expressed lower levels of mRNA for all five genes (Fig. 4). Δ*gna-1* strains exhibited an 8-fold decrease in *cbh-1* transcripts, >10-fold differences in *gh6-2* and *gh5-1*, a 4-fold decrease in *cdh-1*, and a 7-fold decrease in *gh3-4*. Δ*gna-3* strains had 3-fold reductions of mRNA for *cbh-1* and *gh6-2* and 2-fold decreases in *gh5-1*, *cdh-1*, and *gh3-4*. Δ*gnb-1* strains had a 3-fold decrease in *cbh-1* mRNA levels and 5-fold decreases in both *gh6-2* and *gh5-1*. In addition, this strain had no detectable message for *cdh-1* or *gh3-4* (Fig. 4D and E). Δ*cr-1* mutants had a 2-fold decrease in expression of *cbh-1* but normal levels of *gh6-2*, *gh5-1*, *cdh-1*, and *gh3-4*. This implies that transcription of these four genes may be influenced by a non-cAMP-related target of G-protein signaling, since their mRNA levels are also reduced in Δ*gna-1*, Δ*gna-3*, and Δ*gnb-1* mutants. Δ*gna-2* and Δ*cpc-2* mutants had wild-type levels of mRNA for most genes, with the exception of *cdh-1* and *gh3-4*, which were expressed at levels 3- to 5-fold higher in Δ*cpc-2* mutants.

The findings from qRT-PCR analysis further support a positive regulatory role for GNA-1 and GNA-3 in the control of cellulase activity. The low levels of mRNA for the five genes observed in Δ*gna-3* and Δ*gnb-1* mutants may be influenced by the apparent absence of GNA-1 protein in both strains (Fig. 2). *cpc-2* and *cr-1* appear to have posttranscriptional effects on the expression of cellulase genes, since the corresponding mutants lack cellulase activity (Fig. 1). Since the proteins appear to be made in these mutant backgrounds (Fig. 1 and S3), these may affect posttranslational modifications required for the cellulase enzymes to be functional (47, 48). It should be noted that since the time point selected for our study was early during cellulase induction, it is possible that levels of these mRNAs may be higher at later times after transfer to Avicel.

### cAMP supplementation results in full rescue of Δ*gna-3* mutants and partial remediation of Δ*gna-1* and Δ*gnb-1* strains.

Δ*gna-1*, Δ*gna-3*, Δ*gnb-1*, and Δ*cr-1* mutants exhibit defects in cAMP signaling on VM-sucrose (21, 26, 30, 49), and we have previously shown that the defects of Δ*cr-1* and Δ*gna-3* mutants are partially corrected by exogenous cAMP. We investigated whether this was the case on VM-Avicel by supplementing the most affected strains with cAMP: Δ*gna-1*, Δ*gna-3*, *gna-3^Q208L^*, Δ*gnb-1*, Δ*cpc-2*, and Δ*cr-1*. Exogenous cAMP should bypass the missing signaling components and directly activate PKA (29).

**FIG 5:**
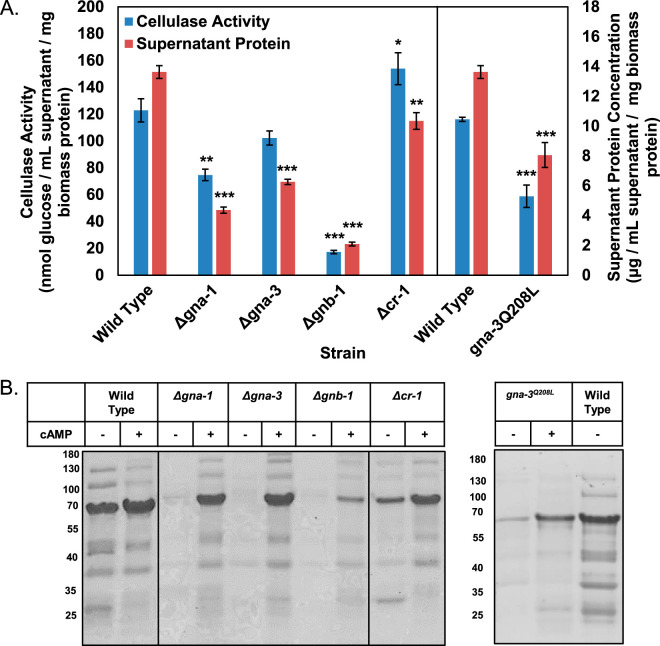
Analysis of strains with cAMP added to the medium. Cultures were grown as described in the legend to Fig. 1, with the exception that cAMP was added to VM-glucose and VM-Avicel at a concentration of 3 mM. (A) Cellulase activity and supernatant protein concentrations. Assays for cellulase activity and supernatant protein concentrations were performed as described in the legend to Fig. 1. Errors are expressed as the standard errors. *, *P* < 0.05; **, *P* < 0.01; ***, *P* < 0.001 versus wild-type *mat***a** with cAMP. The vertical line on the graph indicates that the two groups of strains were analyzed separately. (B) SDS-PAGE analysis of supernatants. SDS-PAGE of supernatant proteins from the indicated strains ± added cAMP was performed as described in the legend to Fig. S3, with equal volumes of concentrated protein loaded on the gel.

cAMP supplementation resulted in higher cellulase activity and extracellular protein levels in the wild type, consistent with a positive role for cAMP signaling (Fig. 5A). cAMP led to at least partial remediation in the other six strains (Fig. 5A). Δ*cr-1* mutants had cellulase activity greater than that of wild type, and Δ*gna-3* mutants were restored to wild-type levels. Although cAMP did not fully correct phenotypes in Δ*gna-1*, *gna-3^Q208L^*, and Δ*gnb-1* strains (73%, 17%, and 53% of wild type, respectively) (compare Fig. 1 and Fig. 5A), no cellulase activity was detectable in these strains without exogenous cAMP (Fig. 1). Furthermore, the partial complementation using cAMP observed in these three strains supports a cAMP-independent pathway that contributes to cellulase activity.

Addition of cAMP led to increased extracellular protein levels for Δ*gna-3*, Δ*cr-1*, and *gna-3^Q208L^* strains, while a decrease was observed in Δ*gna-1* and Δ*gnb-1* mutants (see Fig. S4 for comparison of supernatant protein concentration with or without cAMP). SDS-PAGE analysis of supernatant samples demonstrated that cAMP supplementation restored many of the bands present in the wild type to Δ*gna-1*, Δ*gna-3*, *gna-3^Q208L^*, Δ*gnb-1*, and Δ*cr-1* strains (compare Fig. S3A to Fig. 5B).

10.1128/mBio.02419-20.7 FIG S4 Comparison of protein levels between strains with and without exogenous cAMP. Data were taken from Fig. 1 and Fig. 5A. The supernatant protein concentration from Fig. 5A was divided by the supernatant protein concentration in Fig. 1. Errors are expressed as the standard errors. Asterisks indicate a statistically significant difference between supernatant concentration for each strain with and without cAMP added to the growth medium as follows: *, *P* < 0.05; **, *P* < 0.01; ***, *P* < 0.001. Download FIG S4, PDF file, 0.05 MB. Copyright © 2020 Collier et al. 2020 Collier et al. This content is distributed under the terms of the Creative Commons Attribution 4.0 International license.

**FIG 6:**
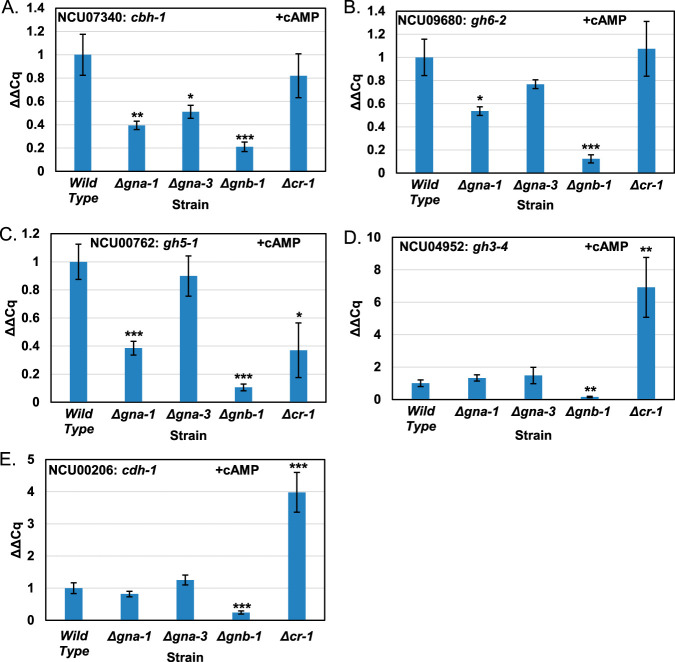
qRT-PCR to determine mRNA levels for major cellulases after growth in Avicel medium containing cAMP. Cultures were grown on VM-glucose containing 3 mM cAMP for 12 h and then transferred to VM-Avicel containing 3 mM cAMP for 4 h. Total mRNA was isolated as described n Materials and Methods. qRT-PCR was carried out as described in the legend to Fig. 5, using RNA isolated from mutants cultured in the presence of cAMP and wild type without cAMP. *, *P* < 0.05; **, *P* < 0.01; ***, *P* < 0.001 versus wild type.

We next asked whether cAMP supplementation restored expression of the five cellulase genes analyzed in Fig. 4 in Δ*gna-1*, Δ*gna-3*, Δ*gnb-1*, and Δ*cr-1* strains. We again used a time point early after transfer to Avicel (4 h). Due to the increased cellulase activity observed in the wild type after cAMP treatment, we utilized the wild type without supplementation as the control. The results showed that Δ*gna-1* strains had increased levels for all five cellulase mRNAs, with those for *cdh-1* and *gh3-4* restored to that of unsupplemented wild type (compare Fig. 4 to Fig. 6). Levels of four mRNAs in Δ*gna-3* mutants resembled those in the wild type, and *cbh-1* levels were increased over those in cultures without added cAMP (Fig. 6). cAMP addition did not restore cellulase mRNA levels in Δ*gnb-1* mutants to those in the wild type but did result in detectable amounts of *cdh-1* and *gh3-4* mRNAs (Fig. 6). Transcript levels for Δ*cr-1* strains were similar to the wild type for *cbh-1*, and levels of *cdh-1* and *gh3-4* were elevated (Fig. 6).

Some hypotheses can be derived from these results (see below) that must be tempered by the difference in timing of the samples used for qRT-PCR analysis and measurement of cellulase activity. Since loss of *gna-3* also leads to greatly reduced levels of GNA-1 protein (Fig. 2), effects due to loss of either Gα subunit cannot be easily resolved using a Δ*gna-3* mutant. Despite low levels of GNA-1, exogenous cAMP increased early cellulase transcription in the Δ*gna-3* mutant, consistent with cAMP acting downstream of both Gα subunits and bypassing the negative regulatory effect of GNB-1 on GNA-3. Loss of *gnb-1* also impacts early cellulase transcription, perhaps due to the effect that *gnb-1* has on GNA-1 and GNA-3 protein levels (Fig. 2). Exogenous cAMP did not affect the Δ*gnb-1* mutant as much as either Gα single mutant, indicating that GNB-1 may also possess a positive regulatory role, independent of cAMP signaling. Finally, despite the posttranscriptional role adenylyl cyclase (*cr-1*) appears to play in regulating cellulase activity (compare Fig. 1 and 4), exogenous cAMP increases cellulase transcription in the Δ*cr-1* mutant (Fig. 6).

## DISCUSSION

**FIG 7:**
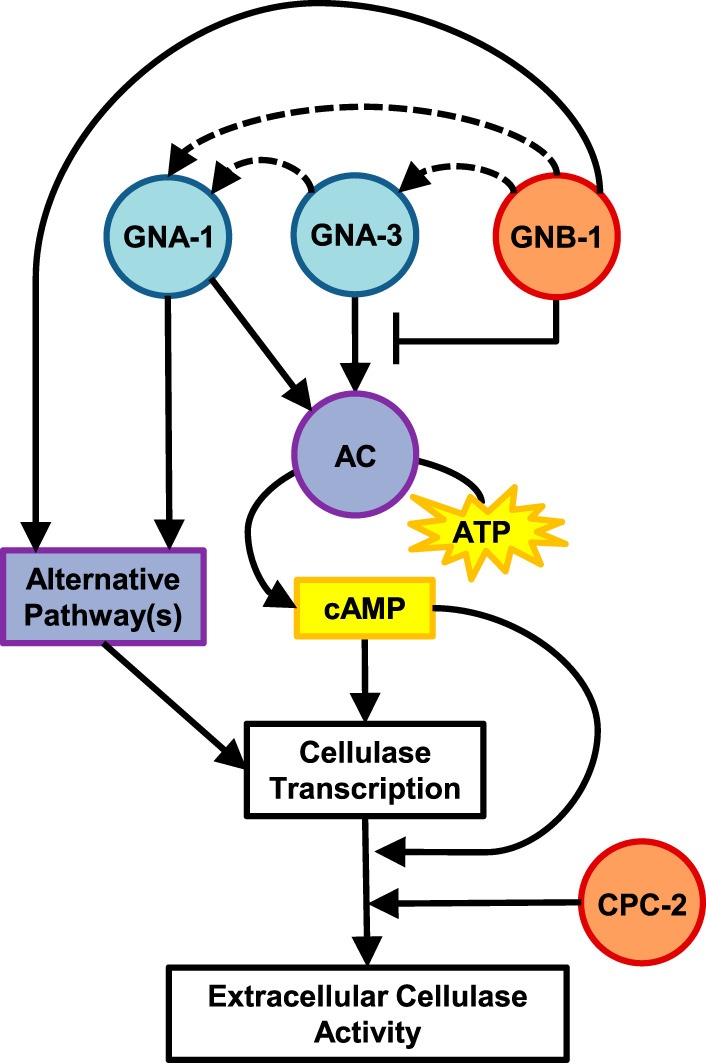
Model for regulation of cellulase activity by G-protein signaling. Gα proteins are shaded in blue, Gβ subunits in orange, effector pathways in purple, and small molecules in yellow. Positive regulation is indicated by solid or dashed lines with arrowheads, while negative regulation is shown using a line that ends with a perpendicular line. Dashed arrows illustrate effects on protein levels. The Gα proteins GNA-1 and GNA-3 positively regulate cellulase activity via cAMP levels through adenylyl cyclase (AC/CR-1). G-protein signaling also controls cellulase transcription via an unknown effector pathway(s) that may correspond to one or more of the three MAPK cascades, Ca^2+^/calmodulin signaling, or another mode of signal transduction. GNA-3 and the Gβ subunit GNB-1 are required to maintain GNA-1 protein levels (indicated by the dashed arrows). Activated GNA-1 is able to overcome the negative regulation by GNB-1 on cellulase production, while the positive role of GNA-3 is only manifested in the absence of GNB-1. AC/CR-1 and the RACK1 Gβ homolog CPC-2 play posttranscriptional roles in regulation of cellulase activity/supernatant protein levels.

We used a comprehensive approach to investigate the effect that every G protein subunit has on cellulose metabolism in a filamentous fungus. We measured extracellular cellulase activity and protein concentration, levels of several cellulase mRNAs, and amounts of each G protein subunit in the strains. Our findings support a scenario in which *gna-1*, *gna-3*, *gnb-1*, and *gng-1* regulate cellulase activity, partially due to their influence on cAMP signaling (see model in Fig. 7). Our results show that GNA-1 and GNA-3 are the major positive regulators of cellulase transcription via cAMP signaling in N. crassa. Additionally, the phenotypes observed in some mutants can be explained by the combined effect of the mutation and reduced levels of other G protein subunit(s). For example, *gnb-1* is required to maintain normal levels of GNA-1 and GNA-2 during growth on Avicel medium, and loss of *gna-3* also resulted in lower levels of GNA-1 protein. The positive effect of GNA-3 was not observed until GNA-3 was constitutively activated and *gnb-1* was absent, demonstrating that GNB-1 is a negative regulator of GNA-3. We suggest that there is a tethering relationship between the Gβγ dimer and GNA-3 that may inhibit signaling to downstream effectors.

Exogenous cAMP rescued phenotypes observed in Δ*cr-1* mutants, bypassing the loss of adenylyl cyclase and activating PKA. Δ*cr-1* mutants had decreased levels of mRNA for only one of the five cellulase genes analyzed, indicating that *cr-1* has a minimal effect on transcription 4 h after transfer to Avicel. This suggests that the CR-1 protein mainly regulates cellulase activity through a posttranscriptional mechanism, with the caveat that cellulase activity was measured using a later time point than for the mRNA levels. The observation that cAMP addition results in partial rescue of cellulase phenotypes in Δ*gna-1* and Δ*gna-3* mutants supports a mechanism in which the GNA-1 and GNA-3 Gα proteins modulate cellulase transcription through adenylyl cyclase. However, early transcription is more impaired in Δ*gna-1*, Δ*gna-3*, and Δ*gnb-1* mutants than in the Δ*cr-1* mutant, and cAMP did not completely remediate Δ*gna-1* or Δ*gnb-1* phenotypes. These results suggest that G proteins may also regulate cellulase transcription through additional pathways not related to cAMP signaling.

There are several possibilities for the alternative pathway(s) downstream of G-protein signaling that could regulate cellulase gene transcription. There is precedence for involvement of mitogen activated protein kinase (MAPK) cascades in cellulase regulation in fungi. For example, the osmosensing (OS) MAP kinase cascade in N. crassa suppresses cellulase expression during conditions when free carbohydrates are available (higher osmolarity) (10). Under low osmolarity (i.e., growth on an insoluble carbon source), this pathway leads to cellulase induction. Results from transcriptome sequencing (RNA-seq) analysis of *T. reesei* strains lacking the *tmk2* MAPK grown on sugarcane baggase revealed that genes involved in G-protein-related processes are differentially regulated in this background. Among these are the *T. reesei* GNA-1 homolog as well as three Pth11-like GPCRs and an RGS gene (50), directly tying G-protein signaling to this MAPK pathway.

Other studies in *T. reesei* implicate calcium signaling in cellulase gene expression. Addition of 50 to 100 mM Ca^2+^ to Avicel medium resulted in higher levels of cellulase activity and biomass production in the hypersecreting Rut-C30 strain (51), and loss of the transcription factor gene *crz1*, a downstream target of the Ca^2+^/calmodulin pathway, resulted in reduced cellulase activity and transcription of *cbh1*, *eg1*, and *xyr1*. *crz1* was found to bind to the promoter of *cbh1*, and its binding affinity increases with added calcium (51). The homologous components of the Ca^2+^/calmodulin pathway in N. crassa have been characterized (52, 53) and may also play a role in regulating cellulase expression.

Our work supports a posttranscriptional role for CPC-2 in the control of cellulase activity in N. crassa. Cellulase mRNA levels in the Δ*cpc-2* mutant are either similar to or greater than those in the wild type, but the Δ*cpc-2* mutant lacks detectable cellulase activity and does not respond to cAMP. We propose that CPC-2 may impact posttranslational modifications of cellulases required for activity, perhaps through translational control of the modifying proteins (47, 48). Additional experiments are necessary to determine the exact posttranscriptional effect of CPC-2 on cellulase production in N. crassa.

Previous work has determined the transcription factors responsible for cellulase induction (10, 34), the intracellular molecule that induces cellulolytic enzymes (54), and the adaptor proteins needed for efficient exit of cellulases from the endoplasmic reticulum (ER) to the Golgi apparatus in fungi (48). Our results contribute to this body of knowledge by providing a comprehensive analysis illustrating the importance of G-protein signaling during upstream regulation of cellulose sensing and cellulase induction. Further study will be required to characterize the detailed molecular mechanism(s) underlying this relationship as well as to determine whether the actions of various G protein subunits influence cellulase mRNA transcription, stability, or translation or regulate posttranslational modification or the secretion of cellulases in filamentous fungi.

## MATERIALS AND METHODS

### Media and strains.

The strains used in this study are indicated in Table S1 in the supplemental material, and strain construction is described in Text S1. Strains were cultured on Vogel’s minimal medium (VM) (55), with the exception that the carbon source indicated in Results replaced sucrose. All carbon sources were used at a final concentration of 2% (wt/vol). Alternative carbon sources were crystalline cellulose (Avicel-PH101, 50-μm particle; Sigma-Aldrich, St. Louis, MO), glucose (MP Biomedicals, Santa Ana, CA), or no carbon source. Where indicated, cAMP (Sigma-Aldrich, number A6885) was added to the medium at a final concentration of 3 mM after autoclaving. Macroconidia (conidia) or packed hyphae used for inoculation of cultures were obtained as described previously (25).

### Isolation of culture supernatants, cellulase and protein assays, and Western blot analysis.

Cultures were inoculated with conidia at a final concentration of 1 × 10^6^ cells/ml in 25 ml VM-glucose and grown for 16 h at 25°C in constant light with shaking at 200 rpm. Cell pads were harvested via centrifugation at 5,000 rpm (Avanti J26XP, JS-5.3 rotor; Beckman Coulter) at 20°C for 5 min, and the supernatant was removed. Cell pads were resuspended in 25 ml VM with no carbon source and centrifuged again. This wash step was repeated once. The cell pads were then resuspended in 25 ml VM-Avicel and transferred to a new sterile flask. The cultures were incubated at 25°C for 3 days in constant light with shaking at 200 rpm. The cultures were then centrifuged at 5,000 rpm at 20°C for 5 min, and the supernatant was separated from the cell pad. Cells were removed from culture supernatants by passage through a 0.45-μm filter, and the filtrate was used for assay of protein concentration and cellulase activity. For SDS-PAGE, 1 ml of supernatant sample was concentrated to 200 μl (Amicon Ultra 4 centrifugal filter unit; MilliporeSigma, Burlington, MA), and 20 μl was loaded on a 10% SDS-PAGE gel. Gels were stained with GelCode Blue (Thermo Fisher Scientific).

A coupled enzyme assay (41) was used to quantify the ability of the culture supernatants to convert cellulose into glucose. Units are expressed as nanomoles of glucose produced per microliter of supernatant. Protein concentration was determined for the supernatant samples using the Pierce bicinchoninic acid (BCA) protein assay (Thermo Fisher Scientific, Chino, CA) with bovine serum albumin (BSA) as the standard. The cellulase activity and protein concentration in the supernatants was normalized to the amount of protein in the cell pad. Extraction of cell pad protein is described in Text S1.

Cell disruption and isolation of whole-cell extracts and the membrane particulate fraction for Western blot analysis is described in Text S1. Equal amounts of protein were electrophoresed using 10% SDS-PAGE gels and blotted to nitrocellulose as previously described (26). Primary antibodies (25, 39, 40, 56, 57) were used at a 1:1,000 dilution. Incubation with secondary antibody and chemiluminescence detection were as previously described (26).

### Total RNA isolation and quantitative reverse transcriptase PCR.

Cultures were grown in VM-glucose for 12 h at 25°C in constant light with shaking at 200 rpm and washed with VM-no carbon as described above. After washing, cell pads were transferred to VM-Avicel and incubated at 25°C for 4 h in constant light with shaking at 200 rpm. Methods for cell collection, mRNA extraction, production of cDNA, and qRT-PCR are described in Text S1. Three biological replicates, each with three technical replicates, were tested for each strain for a total of nine determinations per gene transcript. The primers used for qRT-PCR are listed in Table S2.

10.1128/mBio.02419-20.2 TABLE S2 Primers used in this study. Primers for detection of gene deletion mutants were created as described in reference 13. Primers for qRT-PCR for actin, *cbh-1*, *gh6-2*, and *gh5-1* were as described in reference 14. Primers for qRT-PCR for *cdh-1* and *gh3-4* were designed during this study. Download Table S2, DOCX file, 0.02 MB. Copyright © 2020 Collier et al. 2020 Collier et al. This content is distributed under the terms of the Creative Commons Attribution 4.0 International license.
