# Supplementary material for: Heterotrimeric G-Protein Signaling Is Required for Cellulose Degradation in Neurospora crassa
Source: mBio. 2020 Nov 24;11(6):e02419-20. doi: 10.1128/mBio.02419-20 (PMC7701987; doi:10.1128/mBio.02419-20)
Supplement: TABLE S1 [file mBio.02419-20-st001.docx]

**Table S1. Strains used in this study.**

| **Strain** | **Relevant Genotype** | **Source/**  **Reference #** |
| --- | --- | --- |
| 74-OR23-1A | Wild type, *mat A* | FGSC^1^ |
| OR8-1a | Wild type, *mat a* | FGSC |
| 6103 | *his*-*3, mat A* | FGSC |
| 3b10 | *∆gna*-*1*::*hph, mat a* | (1) |
| 12378 | ∆*gna*-*2*::*hph, mat A* | FGSC |
| 31c2 | ∆*gna*-*3*::*hph, mat A* | (2) |
| 42-8-3 | ∆*gnb*-*1*::*hph, mat A* | (3) |
| 5-5-3 | ∆*gng*-*1*::*hph, mat A* | (4) |
| ∆1gna-1* | ∆*gna*-*1*::*hph, gna*-*1^Q204L^*::*his*-*3^+^, mat A* | This study |
| G2-7 | p*ccg*-*1*::*gna*-*2^Q205L^*::*his*-*3^+^, mat A* | This study |
| gna3Q208L | p*ccg*-*1*::*gna*-*3^Q208L^*::*his*-*3^+^, mat A* | This study |
| cpc-2-5 | ∆*cpc*-*2*::*hph, mat A* | (5) |
| ∆cr-1 | ∆*cr*-*1*::*hph, mat a* | FGSC |
| G1-F | ∆*gnb*-*1*::*hph, his*-*3,* p*ccg*-*1*::*gna*-*1^Q204L^*::*his*-*3^+^, mat a* | (6) |
| G2-D | ∆*gnb*-*1*::*hph, his*-*3,* p*ccg*-*1*::*gna*-*2^Q205L^*::*his*-*3^+^, mat a* | (6) |
| G3-C | ∆*gnb*-*1*::*hph, his*-*3,* p*ccg*-*1*::*gna*-*3^Q208L^*::*his*-*3^+^, mat a* | (6) |

1. FGSC = Fungal Genetics Stock Center (7)

**TABLE S1 REFERENCES**

1. Ivey FD, Yang Q, Borkovich KA. 1999. Positive regulation of adenylyl cyclase activity by a Galphai homolog in *Neurospora crassa*. Fungal Genet Biol 26:48-61.

2. Kays AM, Rowley PS, Baasiri RA, Borkovich KA. 2000. Regulation of conidiation and adenylyl cyclase levels by the Galpha protein GNA-3 in *Neurospora crassa*. Mol Cell Biol 20:7693-705.

3. Yang Q, Poole SI, Borkovich KA. 2002. A G-protein beta subunit required for sexual and vegetative development and maintenance of normal G alpha protein levels in *Neurospora crassa*. Eukaryot Cell 1:378-90.

4. Krystofova S, Borkovich KA. 2005. The heterotrimeric G-protein subunits GNG-1 and GNB-1 form a Gbetagamma dimer required for normal female fertility, asexual development, and Galpha protein levels in *Neurospora crassa*. Eukaryot Cell 4:365-78.

5. Garud A, Carrillo AJ, Collier LA, Ghosh A, Kim JD, Lopez-Lopez B, Ouyang S, Borkovich KA. 2019. Genetic relationships between the RACK1 homolog *cpc-2* and heterotrimeric G protein subunit genes in *Neurospora crassa*. PLoS One 14:e0223334.

6. Won S, Michkov AV, Krystofova S, Garud AV, Borkovich KA. 2012. Genetic and physical interactions between Galpha subunits and components of the Gbetagamma dimer of heterotrimeric G proteins in *Neurospora crassa*. Eukaryot Cell 11:1239-48.

7. McCluskey K, Wiest A, Plamann M. 2010. The Fungal Genetics Stock Center: a repository for 50 years of fungal genetics research. J Biosci 35:119-26.
