# Supplementary figures and images for: Heterotrimeric G-Protein Signaling Is Required for Cellulose Degradation in Neurospora crassa
Source: mBio. 2020 Nov 24;11(6):e02419-20. doi: 10.1128/mBio.02419-20 (PMC7701987; doi:10.1128/mBio.02419-20)

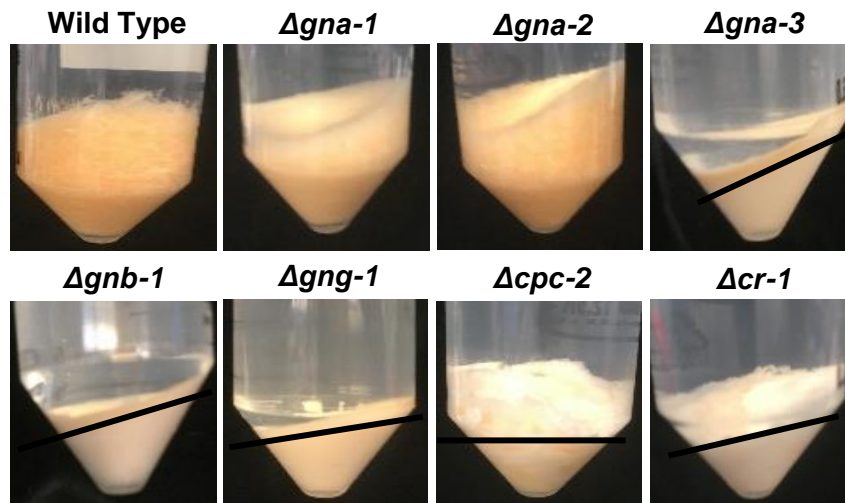

Supplement: FIG S1 [file mBio.02419-20-sf001.pdf]

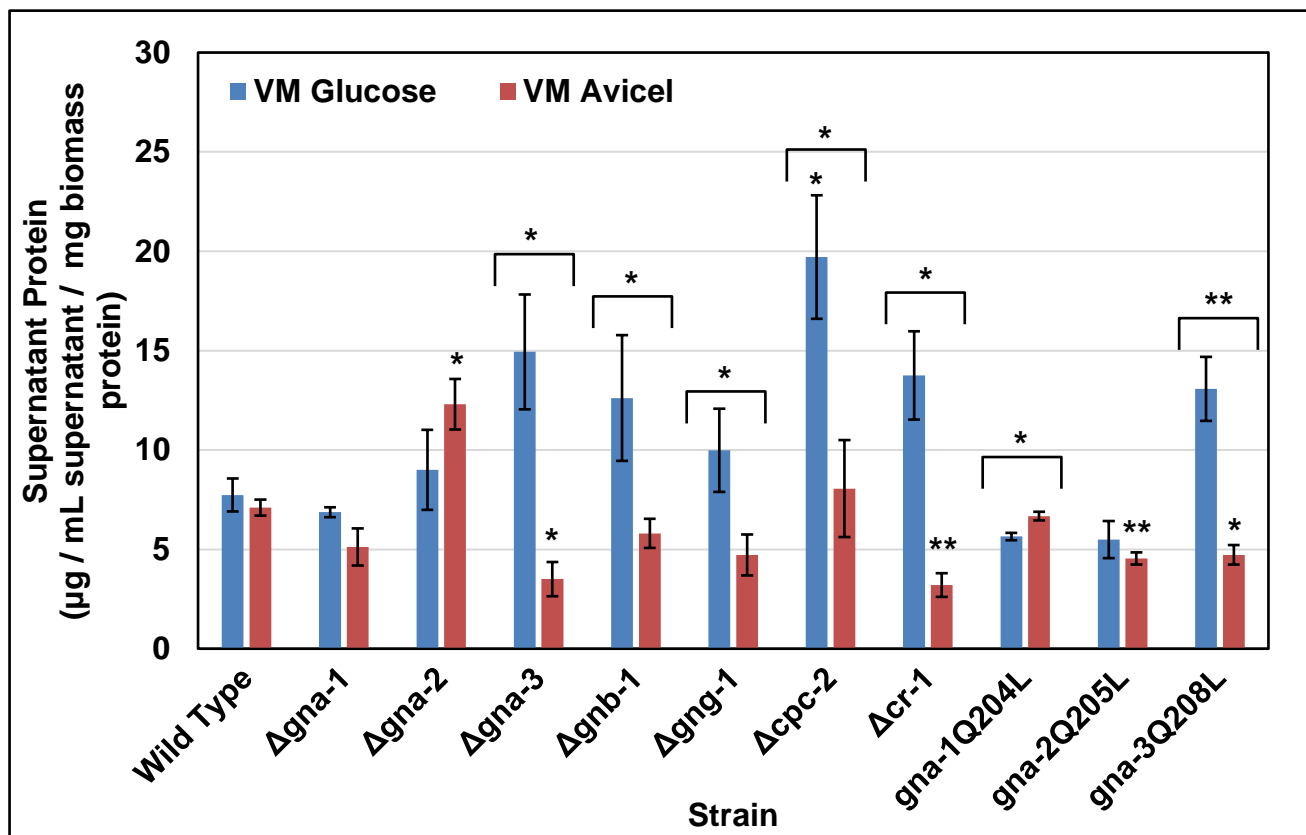

Supplement: FIG S2 [file mBio.02419-20-sf002.pdf]

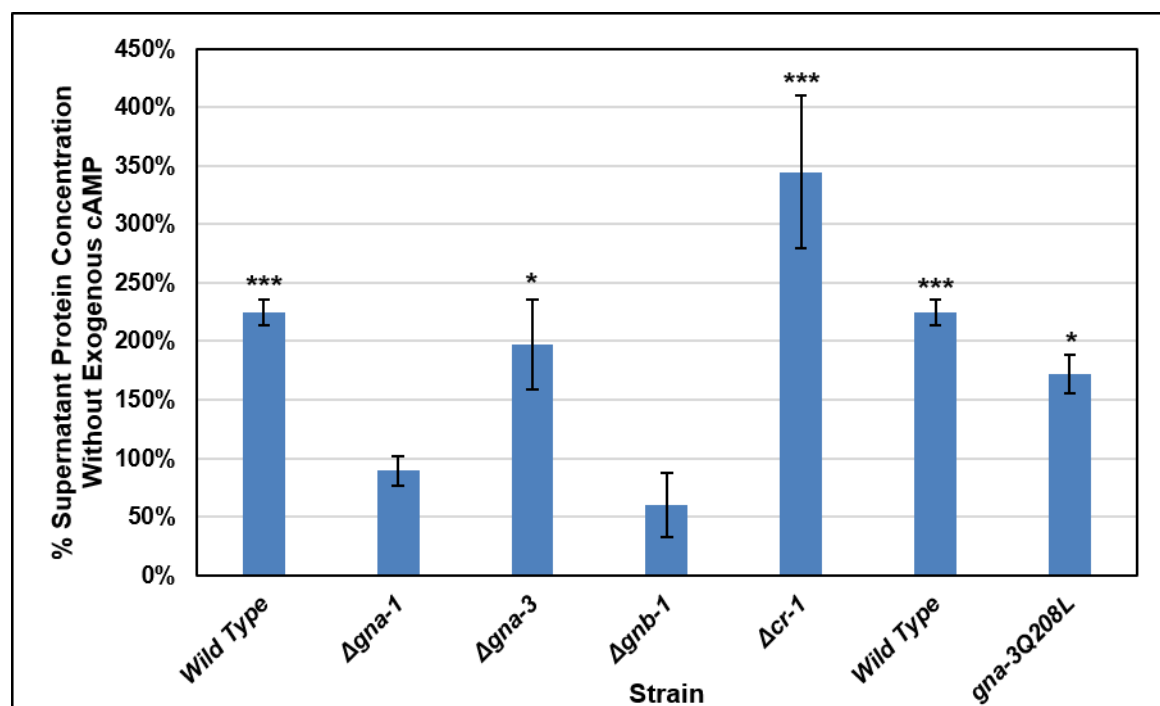

Supplement: FIG S4 [file mBio.02419-20-sf004.pdf]
