# Supplementary material for: Heterotrimeric G-Protein Signaling Is Required for Cellulose Degradation in Neurospora crassa
Source: mBio. 2020 Nov 24;11(6):e02419-20. doi: 10.1128/mBio.02419-20 (PMC7701987; doi:10.1128/mBio.02419-20)
Supplement: TEXT S1 [file mBio.02419-20-s0001.docx]

**SUPPLEMENTAL METHODS**

**Strain construction.** Vectors containing predicted GTPase-deficient, constitutively activating mutations for *gna-1*^Q204L^, *gna-2*^Q205L^, and *gna-3*^Q208L^ (pQY21, pSVK52, and pSVK53, respectively) were previously made using site-directed mutagenesis (1, 2). Electroporation of *N. crassa* with 1-2 µg of pQY21, pSVK52 or pSVK53 was as previously described (3), using the FGSC6103 strain (Table 1) as the recipient, with selection on FGS plates without histidine. Genomic DNA was extracted from transformants and subjected to Southern analysis for *gna-1, gna-2* or *gna-3* as described (1). Transformants determined to have a single integration event of the transforming DNA at the *his-3* locus were purified to homokaryons by serial streaking of macroconidia (4) on FGS plates lacking histidine. Genomic DNA was extracted from these strains and analyzed using Southern analysis to confirm that they were homokaryons.

The Δ*cr-1::hph^R^* knockout mutant was deposited at the FGSC as a heterokaryon (FGSC 11514). Homokaryotic mutants were obtained from the heterokaryon after a sexual cross to wild type strain 74-OR23-1VA and plating ascospores on medium containing hygromycin. Progeny were analyzed using diagnostic PCR (4) with *cr-1* and *hph* primers (Table S2; primers designed as in (5)), and then spot-tested on phosphinothricin to check for the presence of the *mus-51* mutation, which is marked with *bar* (6, 7). All other strains were created as described.

**Growth of strains after direct inoculation into avicel medium.** *N. crassa* strains were inoculated at a density of 1 x10^6^ conidia/mL in 25 mL of VM containing 2% Avicel (crystalline cellulose) as the carbon source and grown with shaking in constant light at 25°C for 4 days. Cultures were then centrifuged at 5000xg for 10 min and photographed.

**Extraction of total protein from Avicel cell pads to normalize cellulase activity and supernatant protein level.** The cellulase activity and protein concentration in the supernatants was normalized to the amount of protein in the cell pad from the indicated strains. Cell pads were mixed with 10 ml Protein Extraction Buffer (1% SDS, 50 mM Tris pH 7.5, 5 mM EDTA) and 5 ml glass beads in a 50 ml conical tube and then heated at 60°C with intermittent vortexing over 12 h. The tubes were centrifuged and the supernatant retained and frozen at -20C. This process was repeated 3-4 times. The total extract volume was recorded and the protein concentration determined as described above. The total mg of cell mass protein in each sample was used to normalize cellulase activity and supernatant protein measurements for that biological replicate.

**Discovery proteomics of the 70 kDa region in SDS-PAGE gels of culture supernatants from wild type.** Three biological replicate samples from wild type were subjected to SDS-PAGE and the gels stained using GelCode Blue. The region of the gel containing the 70 kDa bands was excised and destained in 1 mL 50% acetonitrile-water containing 50 mM Triethylammonium bicarbonate (TEAB, Sigma Aldrich, St. Louis, MO) with shaking at 4°C for 16 h. The solution was then removed from the gel slices. Samples were reduced by the addition of 100 μL of 500 mM tris(2-carboxyethyl)phosphine (Thermo Scientific, Rockford, IL) and incubated at 37°C for 1 h. An aliquot containing 50 µL of 500 mM Iodoacetamide (Sigma Aldrich) and 50 µL 50% acetonitrile-water with 50 mM TEAB was subsequently added and samples were incubated in the dark at room temperature for 1 h. After the reducing buffer was removed, 200 µL of 50% acetonitrile-water with 50 mM TEAB was added, and samples were incubated at 4°C for one h with shaking to remove residual staining agent. The solution was discarded and gel pieces were incubated at room temperature with 1mL of ACN for 15 min. The ACN was removed and the gel pieces were dried under nitrogen for 15 min. Gel pieces were reconstituted with the addition of 250 µL of water and 250 µL of 100 mM TEAB. Subsequently, 5 µL (1µg) of trypsin/lysC mix (Promega, Madison, WI) was added and samples were digested at 37°C overnight (~16 h).

An aliquot containing 10 µL of the above digest was injected for liquid chromatography on a Thermo nLC1200 in single-pump trapping mode with a Thermo PepMap RSLC C18 EASY-spray column (2 μm, 100 Å, 75 μm x 25 cm) and a Pepmap C18 trap column (3 μm, 100 Å,  75 μm x 20 mm). Solvents used were A: water with 0.1% formic acid and B: 80% acetonitrile with 0.1% formic acid. Samples were separated at 300 nL/min with a 260 min gradient ,starting at 3% B, increasing to 30% B from 1 to 230 min, then to 85% B at 240 min hold for 10 min, then back to 3% B in 10 min. Mass spectrometry data was acquired on a Thermo Orbitrap Fusion in data-dependent mode. A full scan was conducted using 60k resolution in the Orbitrap in positive mode. Precursors for MS^2^ were filtered by monoisotopic peak determination for peptides, intensity threshold 5.0e3, charge state 2-7, and 60 sec dynamic exclusion after one analysis with a mass tolerance of 10 ppm. Collisionally induced dissociation spectra were collected in ion trap MS^2^ at 35% energy and isolation window 1.6 m/z.

Results were searched individually in Proteome Discoverer 2.2 (Thermo Scientific) against the UniProt FASTA database for *Neurospora crassa* (accessed 2/12/2020). The precursor mass tolerance was set to 10 ppm and fragment mass tolerance to 0.6 Da. Fixed modifications were carbamidomethyl (Cys +57.021 Da), 13C labelled carbamidomethyl (Cys +58.024 Da), and dynamic modifications included methionine oxidation (+15.995 Da) N-terminal acetylation (+42.011 Da). Results were filtered to a strict 1% false discovery rate. The peptide spectral matches (PSMs) for each protein were compared to the total PSMs to give the percentage of the total.

**Preparation of protein extracts for western analysis.** Cultures were grown in VM-Glucose medium in constant light for 16 h with shaking at 200 rpm and washed with VM No Carbon as described in the Methods. After washing, cell pads were transferred to VM Avicel and incubated at 25°C for three days. Cell pads were pulverized in liquid nitrogen using a mortar and pestle. Four mL of extraction buffer (100 mM Tris, pH 7.5, 1 mM EDTA, 1mM PMSF, 1 mM DTT) was added to the pulverized tissue. The mixture was homogenized using a glass Dounce and the unbroken cells were pelleted by centrifugation at 5000 x g for 10 min at 4°C (Avanti J26XP, JS-5.3 Rotor, Beckman Coulter). An aliquot of the supernatant was reserved as the whole cell extract for western blotting using the CPC-2 antibody. The supernatant was then centrifuged at 46,000 x g (Avanti J26XP, JA-25.50 Rotor, Beckman Coulter) to pellet the particulate fraction as described (1). The pellet was resuspended in wash buffer (100 mM Tris, pH 7.5, 10% glycerol, 1mM PMSF, 1 mM DTT) and was considered the particulate fraction. Protein concentration was determined using the BCA assay, and equal amounts of protein were loaded onto SDS-PAGE gels for western analysis, as described in the Methods.

**Total RNA isolation and quantitative reverse-transcriptase-PCR (qRT-PCR).** Cell pads were collected using vacuum filtration, flash-frozen in liquid nitrogen and then ground using a chilled mortar and pestle with liquid nitrogen. Total RNA was extracted from ground samples using the TRIzol reagent (Thermo Fisher Scientific). Each sample was tested for purity and concentration using a Nanodrop spectrophotometer (Nanodrop 2000c, Thermo Fisher Scientific).

For qRT-PCR, total RNA was treated with RQ1 DNAse (Promega Corporation, Madison, WI) and cDNA was synthesized using the Maxima First Strand cDNA Synthesis Kit (Thermo Fisher Scientific). qPCR was performed using the Dynamo HS SYBR Green qPCR kit (Thermo Fisher Scientific) and the CFX Connect Real Time PCR Detection System (BioRad Laboratories, Inc.) according to the manufacturer’s instructions. Actin (NCU04173) was used as a control. Primers are described in Table S2. Primer sequences for actin, *cbh-1*, *gh6-2*, and *gh5-1* are identical to those in (8).

**SUPPLEMENTAL METHODS REFERENCES**

1. Won S, Michkov AV, Krystofova S, Garud AV, Borkovich KA. 2012. Genetic and physical interactions between Galpha subunits and components of the Gbetagamma dimer of heterotrimeric G proteins in *Neurospora crassa*. Eukaryot Cell 11:1239-48.

2. Yang Q, Borkovich KA. 1999. Mutational activation of a Galphai causes uncontrolled proliferation of aerial hyphae and increased sensitivity to heat and oxidative stress in *Neurospora crassa*. Genetics 151:107-17.

3. Ivey FD, Hodge PN, Turner GE, Borkovich KA. 1996. The G alpha i homologue gna-1 controls multiple differentiation pathways in *Neurospora crassa*. Mol Biol Cell 7:1283-97.

4. Ghosh A, Servin JA, Park G, Borkovich KA. 2014. Global analysis of serine/threonine and tyrosine protein phosphatase catalytic subunit genes in *Neurospora crassa* reveals interplay between phosphatases and the p38 mitogen-activated protein kinase. G3 (Bethesda) 4:349-65.

5. Borkovich KA, Alex LA, Yarden O, Freitag M, Turner GE, Read ND, Seiler S, Bell-Pedersen D, Paietta J, Plesofsky N, Plamann M, Goodrich-Tanrikulu M, Schulte U, Mannhaupt G, Nargang FE, Radford A, Selitrennikoff C, Galagan JE, Dunlap JC, Loros JJ, Catcheside D, Inoue H, Aramayo R, Polymenis M, Selker EU, Sachs MS, Marzluf GA, Paulsen I, Davis R, Ebbole DJ, Zelter A, Kalkman ER, O'Rourke R, Bowring F, Yeadon J, Ishii C, Suzuki K, Sakai W, Pratt R. 2004. Lessons from the genome sequence of *Neurospora crassa*: tracing the path from genomic blueprint to multicellular organism. Microbiol Mol Biol Rev 68:1-108.

6. Avalos J, Geever RF, Case ME. 1989. Bialaphos resistance as a dominant selectable marker in Neurospora crassa. Curr Genet 16:369-72.

7. Pall M. 1993. The use of Ignite (basta; glufosinate; phosphinothricin) to select transformants of *bar*-containing plasmids in *Neurospora crassa*. Fungal Genet Newsl 40:57.

8. Znameroski EA, Coradetti ST, Roche CM, Tsai JC, Iavarone AT, Cate JH, Glass NL. 2012. Induction of lignocellulose-degrading enzymes in *Neurospora crassa* by cellodextrins. Proc Nat Acad Sci USA 109:6012-7.
