# Supplementary material for: Heterotrimeric G-Protein Signaling Is Required for Cellulose Degradation in Neurospora crassa
Source: mBio. 2020 Nov 24;11(6):e02419-20. doi: 10.1128/mBio.02419-20 (PMC7701987; doi:10.1128/mBio.02419-20)
Supplement: FIG S3 [file mBio.02419-20-sf003.pdf]

A.

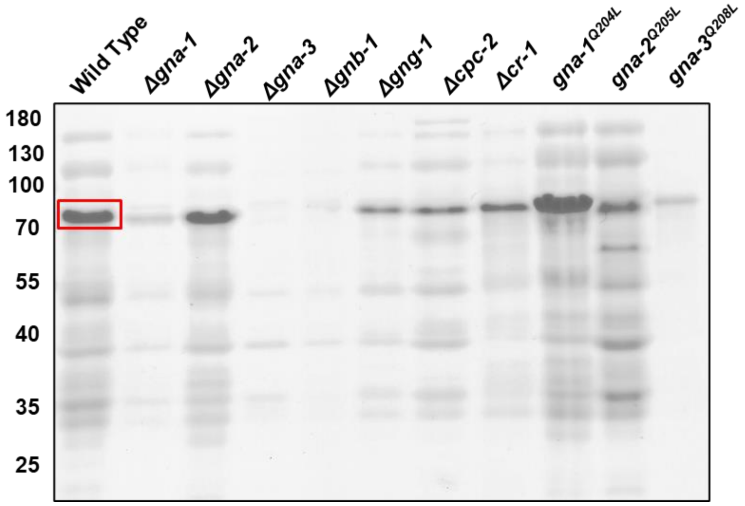

B.

Gene Name	NCU Number	% of total PSMs
cbh-1	NCU07340	41±2%
gh3-4	NCU04952	13±0%
ncw-1	NCU05137	12±2%
gh51-1	NCU02343	6±0%
gla-1	NCU01517	5±1%
inv	NCU04265	5±0%
cdh-1	NCU00206	4±1%
	NCU09024	3±1%
scp	NCU00831	2±0%
mpr-17	NCU09228	2±0%
spr-9	NCU09992	2±0%
gh6-2	NCU09680	2±1%
gel-3	NCU08909	1±0%
gh74-1	NCU05955	1±0%
