# Supplementary material for: Heterotrimeric G-Protein Signaling Is Required for Cellulose Degradation in Neurospora crassa
Source: mBio. 2020 Nov 24;11(6):e02419-20. doi: 10.1128/mBio.02419-20 (PMC7701987; doi:10.1128/mBio.02419-20)
Supplement: TABLE S2 [file mBio.02419-20-st002.docx]

**Table S2. Primers used in this study.**

| **Primer** | **Sequence (5’-3’)^1^** | **Amplicon Size** |
| --- | --- | --- |
| Δ*cr-1* 5’ diagnostic | GGCCCTGGCAGTCAGGTTGC | 1 kb |
| *hph* 5’ diagnostic | GGGATTCATTGTTGACCTCCA |  |
| Δ*cr-1* 3’ diagnostic | TGTCGTCGTCGGTTGGTCGC | 1 kb |
| *hph* 3’ diagnostic | CGCCCCAGCACTCGTCCGAGG |  |
| NCU00206 FWD qRTPCR | TGCTCATCGAGAAGGGTTTC | 90 bp |
| NCU00206 REV qRTPCR | CGAAGCGAGTAAGGGATGTATT |  |
| NCU00762 FWD qRTPCR | GAGTTCACATTCCCTGACA | 72 bp |
| NCU00762 REV qRTPCR | CGAAGCCAACACGGAAGA |  |
| NCU04952 FWD qRTPCR | GAAAGAGCTAGGCTTCAAAGGA | 99 bp |
| NCU04952 REV qRTPCR | CCTGGCATCGTCATATCCATAC |  |
| NCU07340 FWD qRTPCR | ATCTGGGAAGCGAACAAAG | 113 bp |
| NCU07340 REV qRTPCR | TAGCGGTCGTCGGAATAG |  |
| NCU09680 FWD qRTPCR | CCCATCACCACTACTACC | 108 bp |
| NCU09680 REV qRTPCR | CCAGCCCTGAACACCAAG |  |
| Actin FWD qRTPCR | TGATCTTACCGACTACCT | 102 bp |
| Actin REV qRTPCR | CAGAGCTTCTCCTTGATG |  |
